# Survival time analysis in women with breast cancer using distributional regression models

**DOI:** 10.1590/0102-311XEN073324

**Published:** 2025-09-01

**Authors:** Isabela da Silva Lima, Sóstenes Jerônimo da Silva, Carla Regina Guimarães Brighenti, Luiz Ricardo Nakamura, Tiago Almeida de Oliveira, Milena Edite Casé de Oliveira, Thiago Gentil Ramires

**Affiliations:** 1 Programa de Pós-graduação em Estatística e Experimentação Agropecuária, Universidade Federal de Lavras, Lavras, Brasil.; 2 Programa de Pós-graduação em Biometria e Estatística Aplicada, Universidade Federal Rural de Pernambuco, Recife, Brasil.; 3 Departamento de Zootecnia, Universidade Federal de São João del-Rei, São João del-Rei, Brasil.; 4 Departamento de Estatística, Universidade Federal de Lavras, Lavras, Brasil.; 5 Departamento de Estatística, Universidade Estadual da Paraíba, Campina Grande, Brasil.; 6 Departamento de Psicologia, Centro Universitário Tabosa de Almeida, Caruaru, Brasil.; 7 Departamento de Matemática, Universidade Tecnológica Federal do Paraná, Curitiba, Brasil.

**Keywords:** Breast Neoplasms, Mortality, Survival Analysis, Neoplasias de Mama, Mortalidade, Análise de Sobrevida, Neoplasias de la Mama, Mortalidad, Análisis de Supervivencia

## Abstract

Cancer is a global public health concern due to its high mortality rates. In Brazil, breast cancer is one of the leading causes of disease and death among women in all regions of the country, with higher mortality rates in less developed regions. Hence, this study analyzes variables associated with survival time in breast cancer patients in Campina Grande, Paraíba State, Brazil. Distributional regression models, also known as generalized additive models for location, scale, and shape (GAMLSS), were used due to their flexibility in explaining complex behaviors of a given response (for example, survival time) based on other variables. Tumor site, age, number of hormone therapy, radiotherapy and chemotherapy sessions, and molecular markers such as estrogen receptor, progesterone receptor, Ki-67 protein, p53, HER2 mutation and molecular subtype were examined. Two different GAMLSS were fitted considering Weibull and log-normal distributions, the former of which is more appropriate per the Akaike information criterion. Using a variable selection procedure specific to GAMLSS, we identified four covariates that directly affect average survival time: number of hormone therapy and chemotherapy sessions, p53 status, and estrogen receptor status. Excepting estrogen receptor status, the other covariates selected to explain average survival time were also used to explicitly explain the variability of these times.

## Resumo

O câncer é uma preocupação de saúde pública global dada a sua alta taxa de mortalidade. No Brasil, o câncer de mama é uma das principais causas de adoecimento e morte entre mulheres em todas as regiões do país, e as taxas de mortalidade são maiores nas regiões menos desenvolvidas. Portanto, este estudo buscou analisar variáveis associadas ao tempo de sobrevida em pacientes com câncer de mama na região de Campina Grande, no Estado da Paraíba, Brasil. Para isso, foram utilizados modelos de regressão distribucional - também conhecidos como modelos aditivos generalizados para posição, escala e forma (GAMLSS, acrônimo em inglês) - devido à sua flexibilidade em explicar comportamentos complexos de uma determinada resposta (por exemplo, tempo de sobrevida) com base em outras variáveis. Este estudo examinou a localização do tumor, a idade, o número de sessões de terapia hormonal, radioterapia e quimioterapia e marcadores moleculares como receptor de estrogênio, receptor de progesterona, proteína Ki-67, p53, mutação HER2 e subtipo molecular. As distribuições Weibull e *log*-normal ajustaram dois GAMLSS. O critério de informação de Akaike mostrou aquela distribuição como a mais apropriada para análises. Usando um procedimento de seleção de variáveis específico a GAMLSS, identificamos quatro covariáveis que afetam diretamente o tempo médio de sobrevida: número de sessões de terapia hormonal e quimioterapia, status de p53 e *status* do receptor de estrogênio. Com exceção do *status* do receptor de estrogênio, as outras covariáveis selecionadas para explicar o tempo médio também foram usadas para explicar explicitamente a variabilidade desses tempos.

## Resumen

El cáncer es un problema de salud pública mundial porque provoca altas tasas de mortalidad. En Brasil, el cáncer de mama es una de las principales causas de enfermedad y muerte entre las mujeres en todas las regiones del país, y las tasas de mortalidad son más altas en las regiones menos desarrolladas. Por lo tanto, el objetivo de este estudio fue analizar las variables asociadas al tiempo de supervivencia de pacientes con cáncer de mama de la región de Campina Grande, Estado de Paraíba, Brasil. Para ello, se utilizaron modelos de regresión distribucional -también conocidos como modelos aditivos generalizados, para la ubicación, la escala y la forma (GAMLSS, por su sigla en inglés)- debido a su flexibilidad para explicar comportamientos complejos de determinada respuesta (por ejemplo, el tiempo de supervivencia) en función de otras variables. En este estudio se examinó la localización del tumor, la edad, el número de sesiones de terapia hormonal, radioterapia y quimioterapia, y marcadores moleculares como el receptor de estrógenos, el receptor de progesterona, la proteína Ki-67, p53, la mutación HER2 y el subtipo molecular. Se utilizaron dos GAMLSS diferentes considerando las distribuciones de Weibull y *log*-normal, siendo la primera la más apropiada según el criterio de información de Akaike. Utilizando un procedimiento de selección de variables específico para GAMLSS, identificamos cuatro covariables que afectan directamente el tiempo medio de supervivencia: número de sesiones de terapia hormonal y quimioterapia, el estado de p53 y el estado del receptor de estrógeno. Además, a excepción del estado de los receptores de estrógenos, las otras covariables seleccionadas para explicar el tiempo medio también se utilizaron para explicar explícitamente la variabilidad de estos tiempos.

## Introduction

Cancer has become a global public health concern due to its chronic and noncommunicable nature which results in high mortality rates and new cases. Among cancer types, breast cancer is the second most common with an estimated 2.3 million new cases, accounting for 11.6% of all cancer cases. It is the fourth leading cause of cancer-related mortality worldwide, resulting in 666,000 fatalities (6.9% of all cancer deaths). Among female patients, breast cancer is the most frequently diagnosed type and the main cause of cancer-related deaths globally ^1^.

According to the Brazilian National Cancer Institute ^2^, breast cancer is one of the leading causes of disease and death among women in all regions of Brazil. Between 2023 and 2025, approximately 73,610 new breast cancer cases were estimated each year, representing an estimated risk of 66.54 new cases per 100,000 Brazilian women.

Breast cancer onset is associated with several factors such as age, genetic predisposition, lifestyle and environment exposure. One-third of all breast cancer cases worldwide can be successfully treated if diagnosed in early stages ^3^. Less developed regions generally have higher breast mortality rates due to breast cancer ^4^.

Developed regions like Northern and Western Europe show a downward trend in breast cancer mortality ^5^. In developing countries like Brazil, however, the opposite occurs due to behavioral factors, sociocultural obstacles and difficulties in accessing health services. In this context, Northeastern Brazil has seen a serious increase in breast cancer incidence in recent years, from approximately 27 new cases per 100,000 women in 2005 to approximately 52.20 new cases in 2023 ^2^ ^,^ ^6^.

As such, research has shown interest in studying factors related to survival time among breast cancer patients, i.e., which variables influence this time. Survival analysis, which explores the time until the occurrence of a particular event of interest, is an alternative for evaluating the survival probability of individuals under certain conditions. Additionally, by using distributional regression models, firstly introduced as the generalized additive models for location, scale and shape (GAMLSS) ^7^, we can associate patient profile with not only survival time in women diagnosed with breast cancer, but also other parameters of the response distribution.

Thus, this study analyzed variables associated with survival time characteristics among breast cancer patients in Campina Grande, Paraíba State, Brazil, based on the GAMLSS framework.

## Methodology

Our study dataset was obtained from Pereira et al. ^8^ who collected medical records of women with breast cancer treated at the Paraíba Welfare Foundation Hospital (FAP, acronym in Portuguese), a reference hospital for oncology in Campina Grande, between 2005 and 2015, totaling 222 observations. Due to missing data only 105 observations were used in this study, of which 18 were censored. This difference in the total number of observations is due to the removal of medical records missing essential data required to define the variables of interest.

The study was conducted after approval of the Ethics Committee of the Federal University of Campina Grande (CAAE, n. 97198518.9.0000.5182). During data collection, the medical records of breast cancer patients treated at FAP had their personal information anonymized in accordance with Brazil’s General Data Protection Law.

Variables collected included date of first appointment, date of last appointment, date of death, tumor site (right breast, left breast or both), age (in years), number of hormone therapy sessions, number of radiotherapy sessions, number of chemotherapy sessions and molecular markers such as estrogen receptor (positive or negative), progesterone receptor (positive or negative), Ki-67 protein (< 15%, 15-50% or > 50%), p53 mutation (positive or negative), HER2 mutation (positive or negative) and molecular subtype (luminal A, luminal B, HER2 overexpressed or triple negative).

Response variable was the time to patient death from breast cancer, calculated from the date of the first appointment until the date of death, i.e., survival time was calculated from the moment specialized hospital care was initiated. For some patients, however, this data could not be obtained due to study abandonment, end of study follow-up period or death due to another cause. In these cases, the observations were considered censored and are included in the analysis as censored ^9^. Censorship time was calculated from the date of the first appointment to the last visit. The remaining variables were considered as candidates to explain time to death (or censorship) and were chosen based on their availability in the dataset and the results indicating their significance, as reported in the literature ^8^ ^,^ ^10^ ^,^ ^11^ ^,^ ^12^.

Statistically speaking, Cox models are usually applied to survival data ^8^. However, they rely on a strict assumption that hazards are proportional; when this assumption is violated, inferences and consequently result interpretations may not be reliable. To overcome this limitation, some recent works ^13^ ^,^ ^14^ ^,^ ^15^ ^,^ ^16^ ^,^ ^17^ presented GAMLSS as an interesting alternative to model censored data due to its great flexibility allowing for discovery of new relations between data characteristics, patient profiles (explanatory variables) and time to death. Unlike Cox models, this approach accommodates both proportional and non-proportional hazards.

GAMLSS are univariate distributional regression models in which all parameters of the assumed distribution for the response can be modeled as additive functions of explanatory variables. For example, covariates that explicitly affect the median, coefficient of variation, and skewness of the response variable distribution are selected. These models have flexible assumptions, allowing for adjustment of models that accept different distributions for the response variable ^18^.

Mathematically, if *Y* is the response variable, which follows a statistical distribution associated with certain parameters, possibly representing the mean, median, and variability, among other characteristics, GAMLSS ^7^ allows for one or more characteristics (parameters) to be related to observed independent variables using linear or nonlinear smoothing functions. This enables estimating their correct association with the characteristics of the response variable - time to death in this study. Regarding smoothing functions, we adopted *P*-splines ^19^ which are flexible polynomial functions. 

Two distinct two-parameter distributions - Weibull and log-normal distributions - were considered possible candidates in the GAMLSS framework for representing time to death of breast cancer patients density, since they are commonly applied in survival analysis. For the Weibull distribution, *µ* represents the mean of the response distribution and *σ* is a dispersion parameter ^13^. In log-normal distribution, *µ* represents the median and *σ* is also a dispersion parameter ^20^.

Different strategies can be employed to select terms used to explain both parameters of the Weibull and log-normal distributions. In our study, we use a stepwise-based model named Strategy A ^21^. Basically, the model with the lowest Akaike information criterion (AIC) ^22^ value is selected. Interestingly, when a smoothing function is considered to model a covariate we usually evaluate its partial effect using term plots instead of providing a formal test ^15^. For the covariates linearly introduced into the different regression structures, a 10% significance level was adopted to evaluate their statistical relevance. This choice is consistent with studies employing GAMLSS as the predictive model ^13^.

Assessing the adequacy of a fitted GAMLSS typically involves examining worm plots ^23^ generated from normalized quantile residuals ^24^. Basically, if the residuals have a standard normal distribution (mean zero, unit variance and coefficient of skewness and kurtosis equal to zero), then the model is adequate for describing the dataset. 

All statistical analyses were performed using the GAMLSS package ^25^ in R software (http://www.r-project.org).

## Results

First, descriptive and exploratory data analyses were performed. Table 1 summarizes the descriptive statistics for quantitative variables, including the response of patients for whom survival time was available. Time until death distribution showed a slightly positive skewness of 0.39 and a negative kurtosis of -0.50, which indicates a platykurtic distribution with lighter tails. Censored time distribution also exhibited a positive skewed distribution (skewness = 0.29) and a negative kurtosis of -1.09, indicating platykurtic distribution (Table 1).

**Table 1:** Descriptive statistics for the response variable (time to death or censorship) and the quantitative covariates, considering both censored and non-censored observations.

Variable/Group	Mean	Median	Standard deviation	Minimum *	Maximum **	Skewness	Kurtosis
Time (days)							
Non-censored	1,370.20	1,601.00	1,022.69	10.00	4,175.00	0.39	-0.50
Censored	1,050.90	1,064.50	568.99	171.00	2,167.00	0.29	-1.09
Age (years)							
Non-censored	57.83	56.00	12.78	30.00	89.00	0.17	-0.72
Censored	57.00	52.50	14.66	39.00	89.00	0.77	-0.58
Hormone therapy sessions							
Non-censored	36.52	54.00	29.64	0.00	87.00	-0.18	-1.73
Censored	22.33	17.50	20.17	0.00	69.00	0.82	-0.54
Radiotherapy sessions							
Non-censored	26.46	28.00	10.36	0.00	54.00	-0.82	2.66
Censored	31.11	30.00	20.73	0.00	90.00	0.77	1.50
Chemotherapy sessions							
Non-censored	8.29	1.00	14.21	0.00	67.00	2.52	6.47
Censored	20.28	19.50	13.38	0.00	47.00	0.22	-0.58

* Lowest observed value of the data;

** Highest observed value of the data.

Deceased patients has an average age of 58 years. Average time between the initial consultation and patient death was approximately 1,370 days. Additionally, the average number of hormone therapy, radiotherapy and chemotherapy sessions were 36.52, 26.46 and 8.29, respectively.

Regarding censored data, average age was approximately 57 years. Average time between the first appointment and patient censorship was approximately 1,051 days. Average number of hormone therapy, radiotherapy and chemotherapy sessions were 22.33, 31.11 and 20.28, respectively. 

Table 2 presents the frequency of individuals in each group (censored and non-censored) for each categorical covariate under study. Most tumors were detected in the left breast, and a higher frequency of patients exhibited positive estrogen and progesterone receptors. Additionally, most of the sample showed Ki-67 protein below 15%, negative p53 and HER2 mutations, and Luminal B molecular subtype.

**Table 2:** Frequency of individuals in each group (censored and non-censored) for each categorical covariate under study.

Variable/Level	Non-censored frequency	Censored frequency	Total
Tumor site			
Right breast	38	7	45
Left breast	47	10	57
Both	2	1	3
Estrogen receptor			
Positive	76	14	90
Negative	11	4	15
Progesterone receptor			
Positive	64	11	75
Negative	23	7	30
Ki-67 protein (%)			
< 15	45	4	49
15-50	31	10	41
> 50	11	4	15
p53 mutation			
Positive	29	6	35
Negative	58	12	70
HER2 mutation			
Positive	39	5	44
Negative	48	13	61
Molecular subtype			
Luminal A	40	4	44
Luminal B	37	10	47
HER2 overexpressed	5	1	6
Triple negative	5	3	8

After this initial data analysis, two GAMLSS were fitted based on the Weibull and log-normal distributions. For both models, covariate selection used a forward-based strategy employing the AIC value. The final fitted GAMLSS based on the Weibull distribution returned the lowest AIC value of 1,326.87, whereas the log-normal distribution-based model presented an AIC of 1,401.17. Consequently, we selected the Weibull model for further analysis.

**Figure 1:**
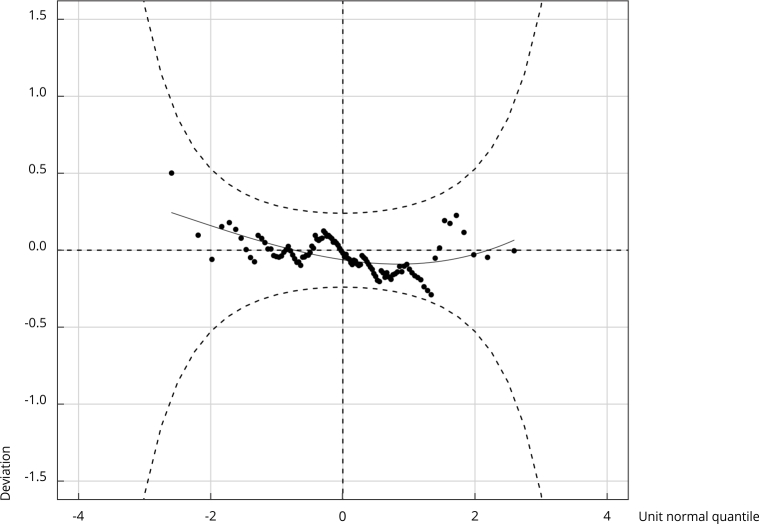
Worm plot for fitted GAMLSS based on the Weibull distribution.

Worm plot (Figure 1) showed that the final model’s assumption, fitted using the Weibull distribution, were met because more than 95% of the residuals were within the 95% confidence intervals (95%CI). The model therefore provided a reasonable fit to these data and was chosen for analysis.

For this final model, the number of hormone therapy sessions (considering a nonlinear smoothing function), number of chemotherapy sessions, estrogen receptor and p53 mutation statuses were selected as covariates to explain the *µ* parameter, i.e., average time to death. Number of hormone therapy sessions (considering a nonlinear smoothing function), number of chemotherapy sessions and p53 mutation status were the variables selected to explain the parameter associated with the variability of *σ*. 

Table 3 summarizes the estimates, standard errors and p-values obtained (except for the variable with a smoothing function) for the final fitted GAMLSS based on the Weibull distribution. All variables were significant at 10% significance level except for p53 status, which was included in the regression structure of the average time to death. From a statistical perspective, however, we kept the variable in the regression structure as excluding variables from the model after a variable selection process such as Strategy A is not reasonable ^26^. 

**Table 3:** Estimates, standard errors and p-values for each of the coefficients from the fitted GAMLSS (generalized additive models for location, scale and shape) based on the Weibull distribution.

	**Mean (*µ*)**			**Variability (*σ*)**		
	Estimate	Standard error	p-value	Estimate	Standard error	p-value
Intercept	6.797	0.122	< 0.001	-0.091	0.166	0.586
Number of hormone therapy sessions	Smoothing function	-	-	Smoothing function	-	-
Number of chemotherapy sessions	0.005	0.001	< 0.001	0.043	0.006	< 0.001
p53 status	0.018	0.032	0.587	-0.825	0.178	< 0.001
Estrogen receptor status	0.220	0.132	0.097			

**Figure 2:**
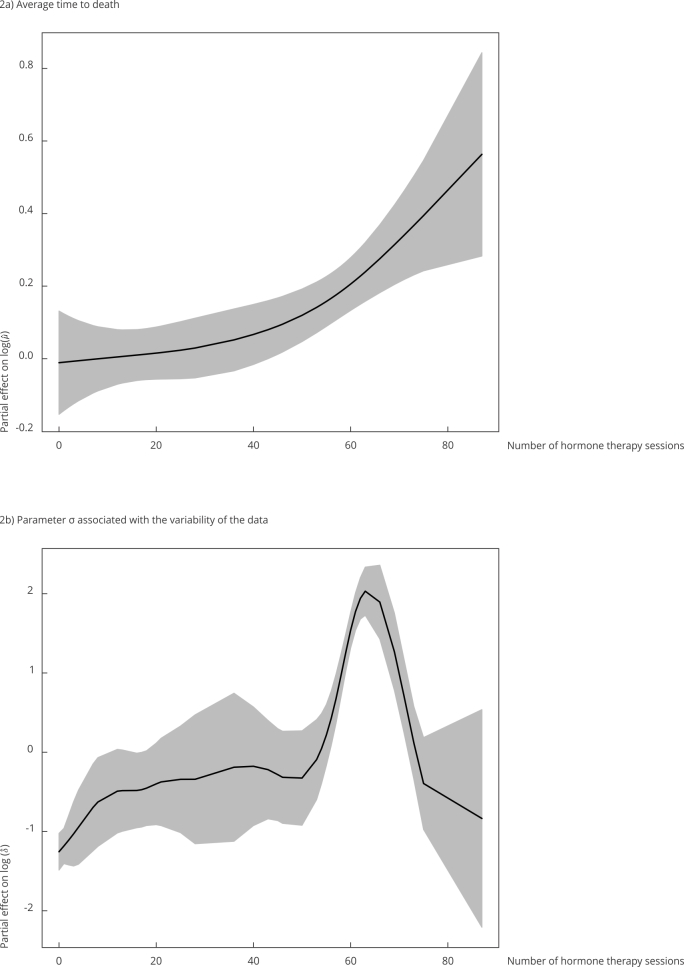
Associations between the number of hormone therapy sessions and average time to death and parameter *σ* associated with data variability.

Regarding the association between the variables used to model average time to death (Table 3), we found the following:

(1) Number of hormone therapy sessions: since we had to include a smoothing function to capture the effect of this covariate on the average time to death, we evaluated it graphically. We found a strictly positive association between this characteristic and time to death (Figure 2a).

(2) Number of chemotherapy sessions: this characteristic was positively related with time to death. More precisely, for each additional session a (exp(0.005 - 1) x 100 = 0.50% increase is expected in a patient’s time to death after the date of the first appointment.

(3) Estrogen receptor (positive or negative): patients presenting estrogen receptor positivity have, on average, a lifespan exp(0.220) = 1.25 times longer (24.61%) after the first consultation compared with those without it. Notably, the p-value associated with this coefficient (0.097) is very close to the adopted significance threshold.

(4) p53 mutation (positive or negative): patients exhibiting p53 expression had, on average, an exp(0.018) = 1.02 (1.82%) longer duration of life after the first consultation than those without it. Importantly, this variable was not significant at the 10% level in the adjusted model.

Based on the associations between the variables used to model the parameter associated with data variability for time to death (Table 3), we found a need to use a smoothing function. As shown in Figure 2b, data variability was practically constant up to 57 sessions, after which variability increased up to approximately 62 sessions before dropping to the same previous level. Moreover, the greater the number of sessions, the greater the variability in the response. In other words, we observed a positive relation between this characteristic and data variability. Finally, patients who lacked p53 mutations had less variability in their time to death.

**Figure 3:**
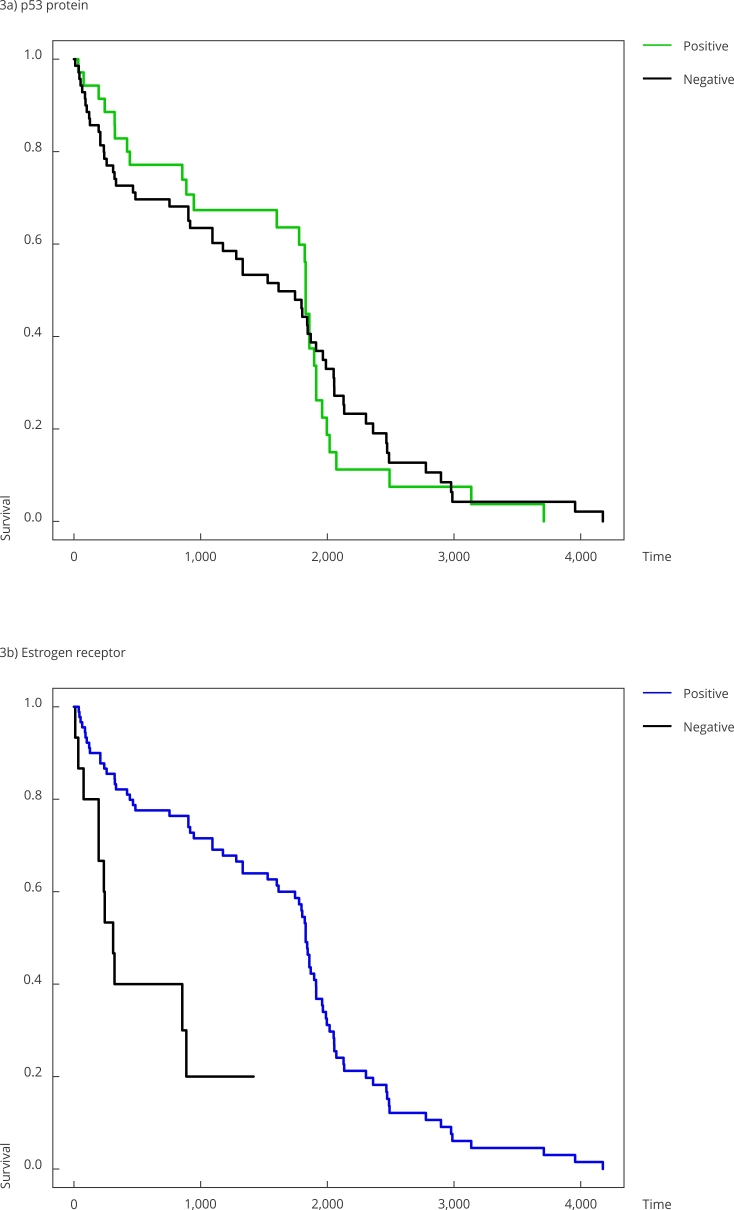
Empirical survival curves (Kaplan-Meier) for p53 protein mutation and estrogen receptor.

Finally, Figure 3 displays the Kaplan-Meier curves stratified by the categorical variables selected for the final fitted model. These empirical curves align with the results in Table 3. 

## Discussion

Statistically, using GAMLSS rather than the traditional Cox models, as in Pereira et al. ^8^, is critical. As seen in Figure 3a, the hazards are clearly not proportional which is the primary assumption of Cox models.

Breast cancer is classified as metastatic or non-metastatic, mostly treated with local therapies which include hormone therapy and chemotherapy ^27^ ^,^ ^28^. Hormone therapy is the main adjuvant systemic for patients who test positive for hormone receptors, lowering estrogen levels or blocking its effects on cancer cells ^29^. In this regard, the literature on breast cancer treatment suggests that administering adjuvant hormone therapy for five years can reduce disease mortality by 40% over a 10-year period compared with women who do not use this therapy, particularly in patients who test positive for hormone receptors ^30^. Additionally, a meta-analysis research found that five years of hormone therapy reduces locoregional recurrence and breast cancer mortality over 10 years ^31^. Our findings are consistent with these studies given the clear positive association between the number of hormone therapy sessions and time to death.

Chemotherapy is one of the most widely used treatments for women affected by breast cancer. It is well established that chemotherapy plays an important role in reducing tumor stage, eliminating micrometastases and alleviating tumor-related symptoms in patients with locally advanced breast cancer ^32^. Advances in chemo efficacy have resulted in increased survival rates for breast cancer patients ^33^. Evidence shows that chemotherapy improves the prognosis for women with breast cancer, with a 17% reduction in the risk of recurrence and a 14% reduction in the risk of death. Consequently, it provides a potential treatment for breast cancer patients, particularly those with regional lymph node involvement ^34^. Again, our results are consistent with previous findings, with each subsequent session resulting in a 0.47% increase in the time to death following the first appointment.

Importantly, patients who live longer may undergo more chemotherapy or hormone therapy sessions, which could affect the observed association between the number of sessions and survival time. This phenomena may reflect a reverse causality bias or a time-dependent covariate bias, as it is unclear whether a higher number of sessions leads to longer survival or if longer survival allows for more treatment sessions. Statistically, the number of sessions changes over time, making it a time-dependent variable ^9^. In conventional survival analysis (without modelling time-dependent factors), patients who die earlier will naturally have undergone fewer sessions, whereas those who live longer will have had time to receive additional treatments, resulting in a correlation that is not necessarily causal.

We thus emphasize that the positive relation between a higher number of sessions and longer survival time may, at least in part, reflect this inherent bias in the retrospective study design. Both factors were included in the analysis to underscore the importance of therapy use (and duration). Nevertheless, prospective studies or specific analyses considering the time-dependent nature of the data are needed to confirm the causal relation.

Our findings about estrogen receptors are also consistent with current research. Breast cancer is considered a heterogeneous disease, with numerous subtypes and cells that have distinct origins and functions ^35^. Its most common type is invasive ductal carcinoma, whereas the most common histological form of non-invasive breast cancer stage is ductal carcinoma *in situ*.

Therapy and survival of breast cancer patients are influenced by the tumor characteristics and the estrogen receptor, progesterone receptor, and HER2 statuses ^36^. Positivity for estrogen and progesterone receptor accounts for most breast cancer. In this regard, the literature suggests that breast tumors with high expression of hormonal receptors are less aggressive and have a better prognosis ^37^ ^,^ ^38^.

Inclusion of p53 protein in the model, even without statistical significance, contributes to a more comprehensive analysis from a clinical and biological perspective. This decision strengthens our interpretations, helps prevent potential biases that could result from removing factors known to be relevant in breast cancer progression, and ensures that its potential effects are not masked or confounded by other covariates in the model.

p53 is a tumor suppressor gene that plays a key role in a variety of cellular mechanisms, including DNA repair, cell cycle regulation, and apoptosis induction ^39^. Previous studies show that the p53 pathway is related with more aggressive diseases and lower overall survival. Thus, depending on the molecular subtype, p53 is observed in 12-84% of breast tumors and is associated with a worse prognosis ^40^ ^,^ ^41^. 

Breast cancer is highly heterogeneous, involving multiple molecular pathways and complex interactions between different biomarkers ^38^. As such, a gene like p53 may not show a clear statistical sign in specific samples or subgroups, but it still contributes to the broader understanding of the disease. Importantly, in studies with relatively small samples, variables with real effects may fail to reach statistical significance. In this context, retaining p53 in the model underscores its clinical relevance, even if the sample size and observed variability were insufficient to statistically confirm its effect. 

Moreover, p53 is referenced in several guidelines and studies as a potential indicator of breast cancer severity. Its inclusion indicates health practitioners and researchers that this component was, at the very least, investigated within the analytical framework, avoiding the omission of variables widely recognized as relevant in oncology literature ^40^. Biologically speaking, it is reasonable to consider this marker in the model, as it could explain significant variations in patient survival. 

Finally, based on the selection process used, the covariates tumor site, age, number of radiotherapy sessions, progesterone receptor, Ki-67 protein, HER2 mutation, and molecular subtype were not included in any of the regression structures. Their effect may potentially already be accounted for by those included in the final fitted model.

## Concluding remarks

GAMLSS were successfully used to analyze survival time data from breast cancer patients undergoing treatment at a hospital in Campina Grande. These findings highlight the versatility of these regression models in various areas of research. Weibull distribution proved to be appropriate for representing the response variable and describing the dataset. The model benefited modeling both the location parameter (average survival time) and the parameter related to time variability, which enabled an objective description and interpretation of the response variable. 

However, the study had some limitations. Absence of clinical data such as stage, tumor size, and histological grade may have hindered results interpretation. Cancer’s biological heterogeneity implies that prognostic factors like tumor aggressiveness and treatment response may have a direct impact on clinical outcomes. To address these limitations, we considered alternative variables to indirectly reflect disease severity and progression, such as the number of chemotherapy, radiotherapy, and hormone therapy sessions, as well as tumor molecular characterization through biomarkers like HER2, Ki-67, and p53 mutation.

Using GAMLSS, which is a more flexible approach than Cox models since it can explain both proportional and non-proportional hazards, allows investigating new relationships between data characteristics and patient profiles. This enables a more comprehensive and adaptive modeling framework than typical parametric regression models. To address the possibility of including strongly correlated variables and avoiding confounding factors, we employed the variable selection method Strategy A which often removes variables with similar explanatory power from the final model.

Although these limitations reduce the precision of prognostic analyses, this study sought to overcome them by incorporating variables related to treatment and tumor biology. Future research should broaden this investigation by using more detailed clinical data and conducting comparative analyses with institutional records.

## References

[1] Bray F, Laversanne M, Sung H, Ferlay J, Siegel RL, Soerjomataram I (2024). Global cancer statistics 2022: GLOBOCAN estimates of incidence
and mortality worldwide for 36 cancers in 185 countries.. CA Cancer J Clin.

[2] Coordenação de Prevenção e Vigilância.Instituto Nacional de Câncer
José Alencar Gomes da Silva (2022). Estimativa 2023: incidência de câncer no Brasil.

[3] Krann R, Colussi CF (2023). Evaluability study of actions for early detection of breast
cancer in primary care. Saude Debate.

[4] Momenimovahed Z, Salehiniya H (2019). Epidemiological characteristics of and risk factors for breast
cancer in the world. Breast Cancer (Dove Med Press).

[5] Carioli G, Malvezzi M, Rodriguez T, Bertuccio P, Negri E, La Vecchia C (2017). Trends and predictions to 2020 in breast cancer mortality in
Europe. Breast.

[6] Coordenação de Prevenção e Vigilância.Instituto Nacional de Câncer
José Alencar Gomes da Silva (2005). Estimativa 2005: incidência de câncer no Brasil.

[7] Rigby RA, Stasinopoulos DM (2005). Generalized additive models for location, scale and
shape. J R Stat Soc Ser C Appl Stat.

[8] Pereira LC, Silva SJ, Fidelis CR, Brito AL, Xavier SFA, Andrade LSS (2022). Cox model and decision trees an application to breast cancer
data. Rev Panam Salud Pública.

[9] Kleinbaum DG, Klein M (2012). Survival analysis: a self-learning text..

[10] El Saghir NS, Seoud M, Khalil MK, Charafeddine M, Salem ZK, Geara FB (2006). Effects of young age at presentation on survival in breast
cancer. BMC Cancer.

[11] Pan Y, Yuan Y, Liu G, Wei Y (2017). P53 and Ki-67 as prognostic markers in triple-negative breast
cancer patients. PLoS One.

[12] Petrelli F, Ghidini A, Antista M, Rossitto M, Dottorini L, Tomasello G (2024). Different prognosis of left compared to right breast cancer a
systematic review and meta-analysis. Cancer Epidemiol.

[13] Ramires TG, Nakamura LR, Righetto AJ, Ortega EMM, Cordeiro GM (2018). Predicting survival function and identifying associated factors
in patients with renal insufficiency in the metropolitan area of Maringá,
Paraná State, Brazil. Cad Saúde Pública.

[14] Ramires TG, Ortega EMM, Hens N, Cordeiro GM, Paula GA (2018). A flexible semiparametric regression model for bimodal,
asymmetric and censored data. J Appl Stat.

[15] Ramires TG, Nakamura LR, Righetto AJ, Pescim RR, Mazucheli J, Cordeiro GM (2019). A new semiparametric Weibull cure rate model fitting different
behaviors within GAMLSS. J Appl Stat.

[16] Ramires TG, Nakamura LR, Righetto AJ, Carvalho RJ, Vieira LA, Pereira CAB (2021). Comparison between highly complex location models and
GAMLSS. Entropy (Basel).

[17] Nakamura LR, Ramires TG, Righetto AJ, Silva V, Konrath AC (2022). Using the Box-Cox family of distributions to model censored data
a distributional regression approach. Braz J Biom.

[18] Stasinopoulos MD, Rigby RA, Heller GZ, Voudouris V, De Bastiani F (2017). Flexible regression and smoothing: using GAMLSS in R.

[19] Eilers PHC, Marx BD (1996). Flexible smoothing with B-splines and penalties. Stat Sci.

[20] Rigby RA, Stasinopoulos MD, Heller GZ, De Bastiani F (2019). Distributions for modeling location, scale, and shape: using GAMLSS in
R.

[21] Ramires TG, Nakamura LR, Righetto AJ, Pescim RR, Mazucheli J, Rigby RA (2021). Validation of stepwise-based procedure in GAMLSS. J Data Sci.

[22] Akaike H (1974). A new look at the statistical model
identification. IEEE Trans Automat Contr.

[23] van Buuren S, Fredriks M (2001). Worm plot a simple diagnostic device for modelling growth
reference curves. Stat Med.

[24] Dunn PK, Smyth GK (1996). Randomized quantile residuals. J Comput Graph Stat.

[25] Stasinopoulos DM, Rigby RA (2007). Generalized additive models for location, scale and shape
(GAMLSS) in R. J Stat Softw.

[26] Lee JD, Sun DL, Sun Y, Taylor J (2016). Exact post-selection inference, with application to the
lasso. Ann Stat.

[27] American Cancer Society Hormone therapy for breast cancer..

[28] Waks AG, Winer EP (2019). Breast cancer treatment a review. JAMA.

[29] Rossi L, Pagani O (2015). The modern landscape of endocrine therapy for premenopausal women
with breast cancer. Breast Care (Basel).

[30] Butani D, Gupta N, Jyani G, Bahuguna P, Kapoor R, Prinja S (2021). Cost-effectiveness of tamoxifen, aromatase inhibitor, and switch
therapy (adjuvant endocrine therapy) for breast cancer in hormone receptor
positive postmenopausal women in India. Breast Cancer (Dove Med Press).

[31] Early Breast Cancer Trialists' Collaborative Group (2015). Aromatase inhibitors versus tamoxifen in early breast cancer
patient-level meta-analysis of the randomised trials. Lancet.

[32] Scholl SM, Asselain B, Palangie T, Dorval T, Jouve M, Giralt EG (1991). Neoadjuvant chemotherapy in operable breast
cancer. Eur J Cancer.

[33] Matsuda T, Takayama T, Tashiro M, Nakamura Y, Ohashi Y, Shimozuma K (2005). Mild cognitive impairment after adjuvant chemotherapy in breast
cancer patients - evaluation of appropriate research design and methodology
to measure symptoms. Breast Cancer.

[34] Zhou X, Tian B, Han HB (2021). Serum interleukin-6 in schizophrenia a system review and
meta-analysis. Cytokine.

[35] Bener A, Barisik CC, Acar A, Özdenkaya Y (2019). Assessment of the gail model in estimating the risk of breast
cancer effect of cancer worry and risk in healthy women. Asian Pac J Cancer Prev.

[36] Parkin DM, Bray F, Ferlay J, Pisani P (2001). Estimating the world cancer burden GLOBOCAN 2000. Int J Cancer.

[37] Khalaf H, Mohammed A, Shukur S, Alhalabi N, Almothafar B, Hassan M (2022). Breast cancer age incidence, hormone receptor status and family
history in Najaf, Iraq. J Med Life.

[38] Sorlie T, Tibshirani R, Parker J, Hastie T, Marron JS, Nobel A (2003). Repeated observation of breast tumor subtypes in independent gene
expression data sets. Proc Natl Acad Sci U S A.

[39] Vogelstein B, Lane D, Levine AJ (2000). Surfing the p53 network. Nature.

[40] Loo LWM, Gao C, Shvetsov YB, Okoro DR, Hernandez BY, Bargonetti J (2019). MDM2, MDM2-C, and mutant p53 expression influence breast cancer
survival in a multiethnic population. Breast Cancer Res Treat.

[41] Cancer Genome Atlas Network (2012). Comprehensive molecular portraits of human breast
tumours. Nature.

